# SoC estimation on Li-ion batteries: A new EIS-based dataset for data-driven applications

**DOI:** 10.1016/j.dib.2024.110947

**Published:** 2024-09-14

**Authors:** Hamza Mustafa, Carmine Bourelly, Michele Vitelli, Filippo Milano, Mario Molinara, Luigi Ferrigno

**Affiliations:** a University of Cassino and Southern Lazio, Cassino, Frosinone, Italy.; b Independent researcher, Italy

**Keywords:** LFP batteries, State of charge, Electrochemical impedance spectroscopy, Dataset

## Abstract

Lithium-ion (Li-ion) batteries are crucial in numerous applications, including portable electronics, electric vehicles, and energy storage systems. Electrochemical Impedance Spectroscopy (EIS) is a powerful technique for characterizing batteries, providing valuable insights into charge transfer kinetics like ion diffusion and interfacial reactions. However, obtaining comprehensive and diverse datasets for battery State of Charge (SoC) studies remains challenging due to the complex nature of battery operations and the time-intensive testing process. This paper presents a novel and original EIS dataset specifically designed for 600 mAh capacity Lithium Iron Phosphate (LFP) batteries at various SoC levels. The dataset includes repeated EIS measurements using different battery discharging cycles, allowing researchers to examine the frequency domain properties and develop data-driven algorithms for assessing battery SoC and predicting performance. The data acquisition system employs a battery specific impedance meter and an electronic load, ensuring accurate and controlled measurements. The dataset, comprising EIS measurements from multiple LFP batteries, serves as a valuable resource for researchers in the fields of battery technology, electrochemistry, power sources, and energy storage. Moreover, industries such as consumer electronics, power systems, and electric transportation can benefit from the dataset's insights for effectively managing rechargeable battery devices. The presented dataset expands the scope of impedance spectroscopy measurements and holds significant potential for future applications and advancements in Li-ion battery technologies.

Specifications Table

**Table:** 

Subject	*Energy Engineering and Power Technology*
Specific subject area	Rechargeable Lithium batteries' Electrochemical Impedance Spectroscopy (EIS), measured at various Stages of Charge.
Type of data	Table
Data collection	The impedance data for the battery, both real and imaginary parts, was measured at frequencies of 0.01, 0.02, 0.03, 0.05, 0.08, 0.1, 0.2, 0.3, 0.5, 0.8, 1, 2, 3, 5, 8, 10, 11, 21, 31, 61, 81, 100, 110, 210, 310, 510, 810, and 1000 Hz. The EIS spectrum was taken for the State of Charge (SoC) levels of 100%, 95%, 90%, 85%, 80%, 75%, 70%, 65%, 60%, 55%, 50%, 45%, 35%, 30%, 25%, 20%, 15%, 10%, and 5%. The measurement was conducted two times on individual discharges of each of the eleven 3.2 V, 600 mAh Lithium Iron Phosphate batteries.
Data source location	Institution: University of Cassino and Southern Lazio, Department of Electrical and Information Engineering City: Cassino Country: Italy Latitude and Longitude: 41.4719°N, 13.8289°E
Data accessibility	Repository name: SoC Estimation on Li-ion Batteries: A New EIS-based Dataset for data-driven applications Data identification number: 10.17632/cb887gkmxw.1 Direct URL to data: https://data.mendeley.com/datasets/cb887gkmxw/1

## 1 Value of the Data

• The obtained data are original and have never before been published in a publication or data repository. The dataset consists primarily of EIS measurement of LFP batteries during the discharge process at various SoC levels. For the proposed dataset, eleven batteries are used; to increase the variability between measurements, the authors try to take the batteries from different batches by buying the cells from different suppliers at different times. The chosen batteries are the 600 mAh LFP made by O'Cell New Energy Technology CO. LTD.
• For each battery, there are two full discharge cycles. The main contribution to the community of the proposed dataset is mainly in the number of used stimulus frequencies (58) and the number of SoC levels (20). To the best of the authors' knowledge, there is no dataset with these two characteristics combined. A high number of stimulus frequencies allows us to study the frequency domain properties of batteries and their variation through the discharge process. In addition, they may be used to create prediction approaches and train machine learning models to assess methods for the reliable and effective management of rechargeable battery devices.
• Researchers in the fields of electrochemical studies and energy storage systems can derive valuable insights from these data, gaining a deeper understanding of cell behavior at different SoC levels. Moreover, they may be beneficial for research and development engineers working on consumer electronics such as IoT devices.

## 2 Background

Currently, batteries represent a highly efficient energy storage means regarding the energy-to-volume ratio and electrical power output. Among the various battery technologies available, Li-ion batteries exhibit exceptional performance in terms of aging, cycle life, and rapid charging capability [1]. Specifically, Lithium Iron Phosphate (LFP) batteries offer unique advantages due to their robust thermal and chemical stability, which provide safety benefits and a longer cycle life compared to other Li-ion chemistries. It has drawn a lot of attention, research, and applications because of its exceptional safety, low cost, low toxicity, and decreased reliance on nickel and cobalt. [2]. LFP batteries are increasingly employed in diverse applications such as portable electronic devices, electric vehicles [3], especially in heavier vehicles due to their safety and life cycle advantages, and stationary energy storage systems where long life and safety are crucial [4].

The characterization of the electrochemical phenomena that occur inside the batteries remains one of the greatest challenges. Despite these advancements, comprehending the electrochemical phenomena occurring within batteries remains a significant challenge. EIS emerges as a potent tool for assessing the performance and degradation processes of Li-ion batteries [5]. EIS measurements on Li-ion batteries apply an alternate signal over various frequencies. The measurements of current and voltage are used to evaluate impedances that could be related to the electrochemical processes occurring within the battery, including charge transfer kinetics, ion diffusion, and interfacial reactions [6]. Those relationships are difficult to address, and many contributions in the literature propose various approaches for the EIS data analysis [7,8].

Impedance spectroscopy methods could be used inside the Battery Management Systems (BMS) to improve the performance in State of Charge (SoC) and State of Health (SoH) estimation. In [9] the authors have demonstrated the possibility of using a classifier to estimate the SoC from the EIS measurements. Furthermore, the impedance spectrum could be used to fit the equivalent electrical circuit models. Those are interesting because the three parts of the spectrum (low-frequency, medium-frequency, and high-frequency) could be used to identify different phenomena occurring in the batteries [10,11]. In this case, having a higher number of frequencies helps the fitting process.

### 2.1 Existing Li-ion batteries datasets

There are some Li-ion battery EIS measurement datasets available publicly. We will discuss these existing datasets briefly.

One of the largest EIS datasets available is published by Zhang et al. [12]. It includes more than 20000 impedance spectra values obtained from 12 Eunicell LR2032 45 mAh LCO/graphite batteries. The cells were cycled at different temperatures after obtaining many frequencies of EIS measurements at various SoC levels, namely 45 °C, 35 °C, and 25 °C. This dataset aims to give data for the batteries' SoH analysis rather than the SoC analysis. For this reason, there are only three impedance spectroscopy during the discharge. The data are provided in “.txt” format and include the EIS values and independent capacity measurements. Another dataset by Buchicchio et al. [13] includes EIS measurements from widely used Li-ion batteries. Using a random-phase multi-sine excitation signal, the batteries' complex impedance was measured at a range of 14 distinct frequencies ranging from 0.05 Hz to 1000 Hz for six batteries. The temperature is kept constant at 25 °C. In this case, there are 10 impedance measurements at as many SoC levels, making this dataset suitable for developing data-driven methods for the SoC evaluation. Despite the novelty of the stimulus signal and the SoC granularity, there are few stimulus frequencies compared with the other dataset. The data are shared in a single “ .csv” format for all the batteries. In [14], the authors share an interesting dataset focused on the charging phase. They tested a 1100 mAh LFP cylindrical battery. The charging phase is a four-step fast charging protocol while the discharge is at a fixed current. The authors tested 240 cells divided into 5 batches. In [15], 34 battery cells from 2 Ah are tested at three different temperatures. This dataset provides the EIS with 39 frequencies from 0.1 Hz to 5 kHz but without giving the specific frequency values. This dataset is focused on the SoH. In [16] 2900 mAh Panasonic 18650PF cell was tested at five controlled environment temperatures from 25 °C to – 20 °C. Each impedance spectroscopy test is performed from 1 mHz to 6 kHz with a step SoC equal to 5%.

## 3 Data Description

**Fig. 1:**
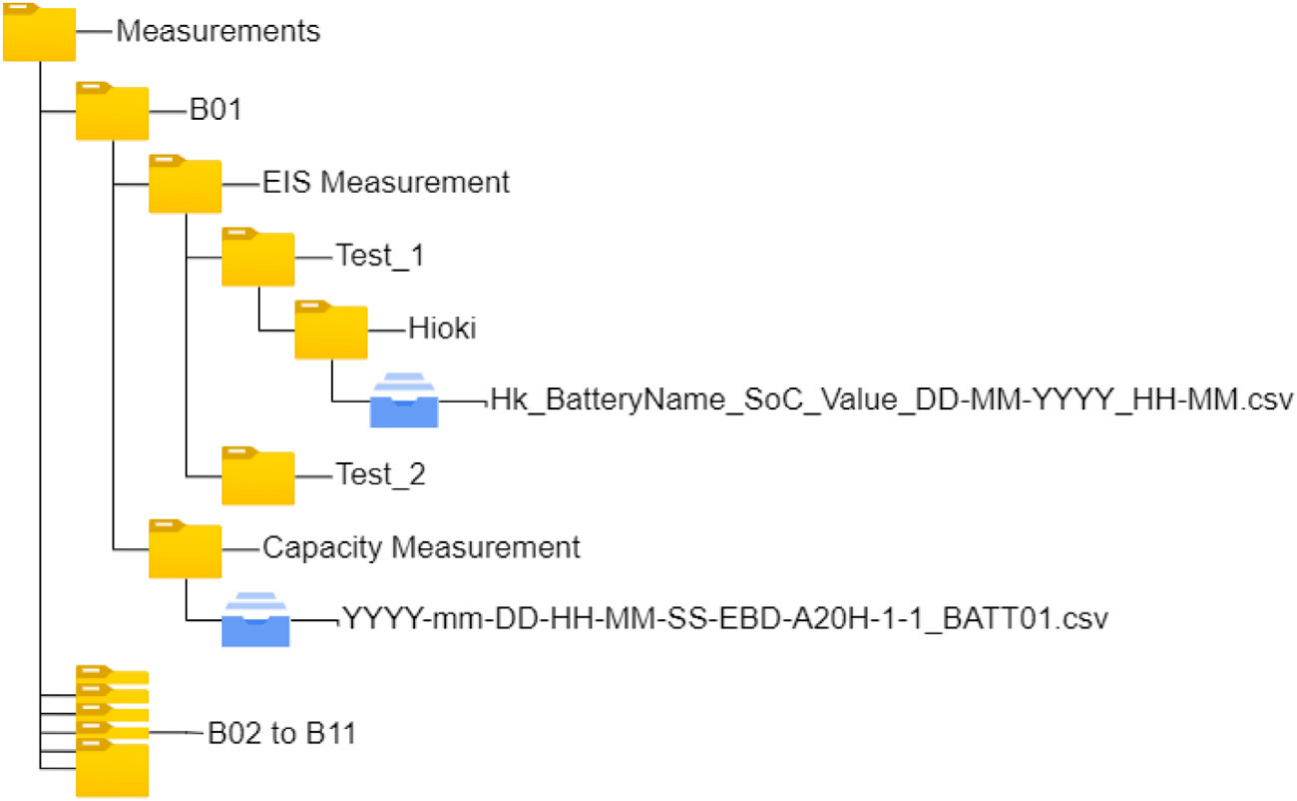
Structure of the obtained experimental dataset.

The dataset is shared with this manuscript. The EIS measurement and Capacity measurement data are recorded in the “.csv” files. These files are organized in directories as shown in Fig. 1, to make them easy to use.

There are eleven directories one for each battery, from “ B01” to “ B11”. Each of these directories further contains two directories, “ EIS Measurement” and “Capacity Measurement”. In the first one there are all the test directories, inside each test folder there are the corresponding test comma-separated values (csv) files with all the impedance spectroscopy measurements. In the second folder, on the same level as “ EIS Measurement” there is the battery capacity test also in a csv formatted file. The following Sections describe in detail the two csv files.

### 3.1 EIS measurements

For each cycle, in the EIS Measurements directory, there is an additional sub-folder named “ Test_N” (where N is the cycle number).

Each Test folder contains the 20 EIS Measurements in the csv files, starting from SoC 5 % to SoC 100 %, with the name starting with the battery model like IFR14500B01. The EIS Measurements files are organized in 6 columns and 28 rows, the following Table 1. describes the columns of these files.

**Table 1:** Measurement file header description.

Output parameters	Column Name	Range	Measuring Unit	Description
Frequency	Frequency	0.01 - 1000	Hz	Frequency used for the Impedance Spectroscopy
Real ($\dot{Z}$)	R	Same of the Range Column	Ω	Real part of the Complex Impedance
Imaginary ($\dot{Z}$)	X	Some of the Range of Column	Ω	Imaginary part of the Complex Impedance
Battery Voltage	V	0 - 20	V	Battery Voltage measured before the Impedance Spectroscopy
Temperature	T	Depends on the climatic chamber	°C	Climatic Chamber temperature setting
Range	Range	0.3 - 3	Ω	Range used for the Impedance Evaluation

### 3.2 Capacity measurements

Under the Capacity evaluation folders, there are the capacity evaluation test files. Each test file reports in the first ten rows the test information like the starting voltage, the voltage, the measured capacity, and the dissipated energy. After three columns are reporting the Time, current, and battery Voltage as shown in the Table 2.

**Table 2:** Measurement file header description.

Column Name	Measuring Unit	Description
Time	s	Time in seconds from the test start to the test end
Cur	A	Discharge current in Ampere
V	V	Battery terminal voltage in Volt

## 4 Experimental Design, Materials and Methods

This Section provides a comprehensive overview of the instruments, connections, and measurement protocols that were specifically developed to obtain the dataset. In contrast to existing datasets documented in the literature, our approach aimed to enhance two key aspects. Firstly, we sought to increase the number of SoC levels included in the dataset, consequently expanding the scope of impedance spectroscopy measurements. Secondly, we aimed to incorporate more frequencies for spectroscopic analysis. This expansion affords significant flexibility within the dataset, making it well-suited for training and testing data-driven algorithms for estimating SoC and investigating the most influential features that correlate SoC with impedance measurements.

### 4.1 Data acquisition system

The adopted measurement setup has the following characteristics:

• the ability to discharge one battery at a time by controlling the discharge current;
• the resolution of SoC measurement lower than 0.1 %;
• the system must also be able to automatically handle the impedance measurement after each step SoC levels ΔSoC.

**Fig. 2:**
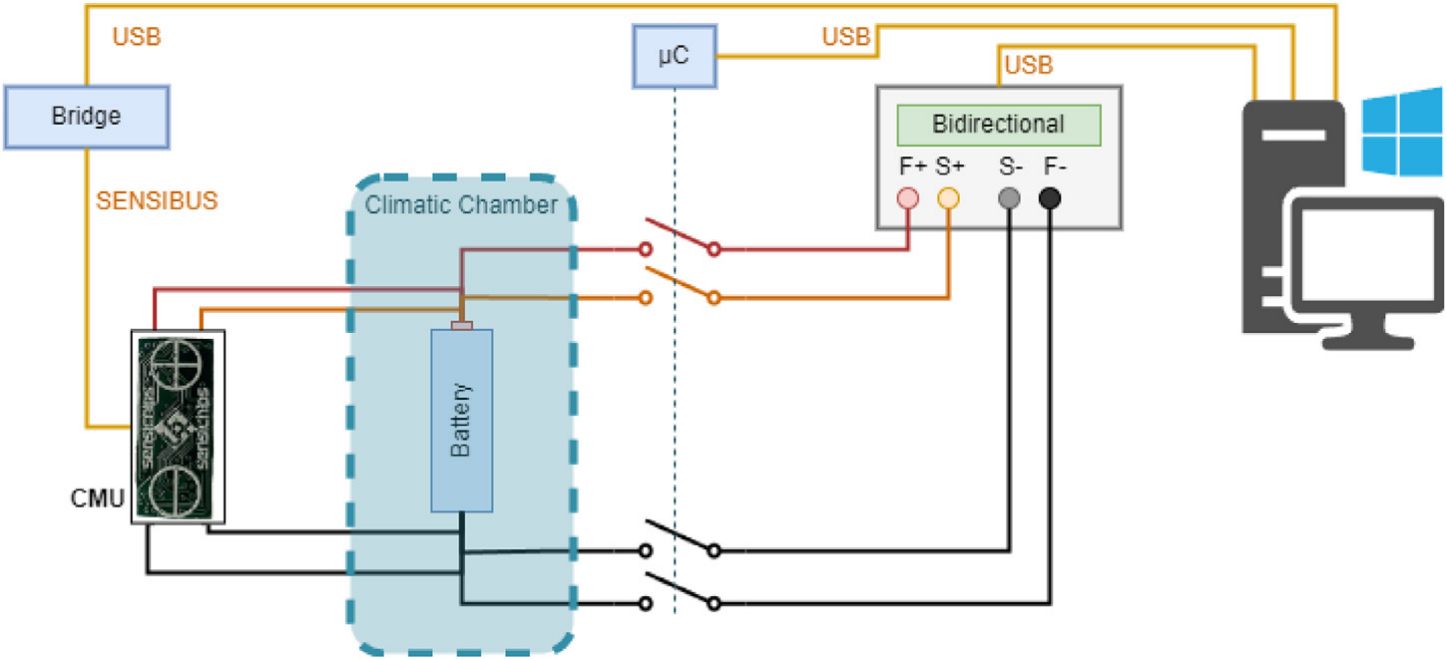
Schematic diagram of the implemented experimental setup.

From the author's experience in designing experimental systems [[17], [18], [19], [20]] the schematic diagram of the implemented experimental setup shown in Fig. 2, is created to meet all the above requirements.

**Fig. 3:**
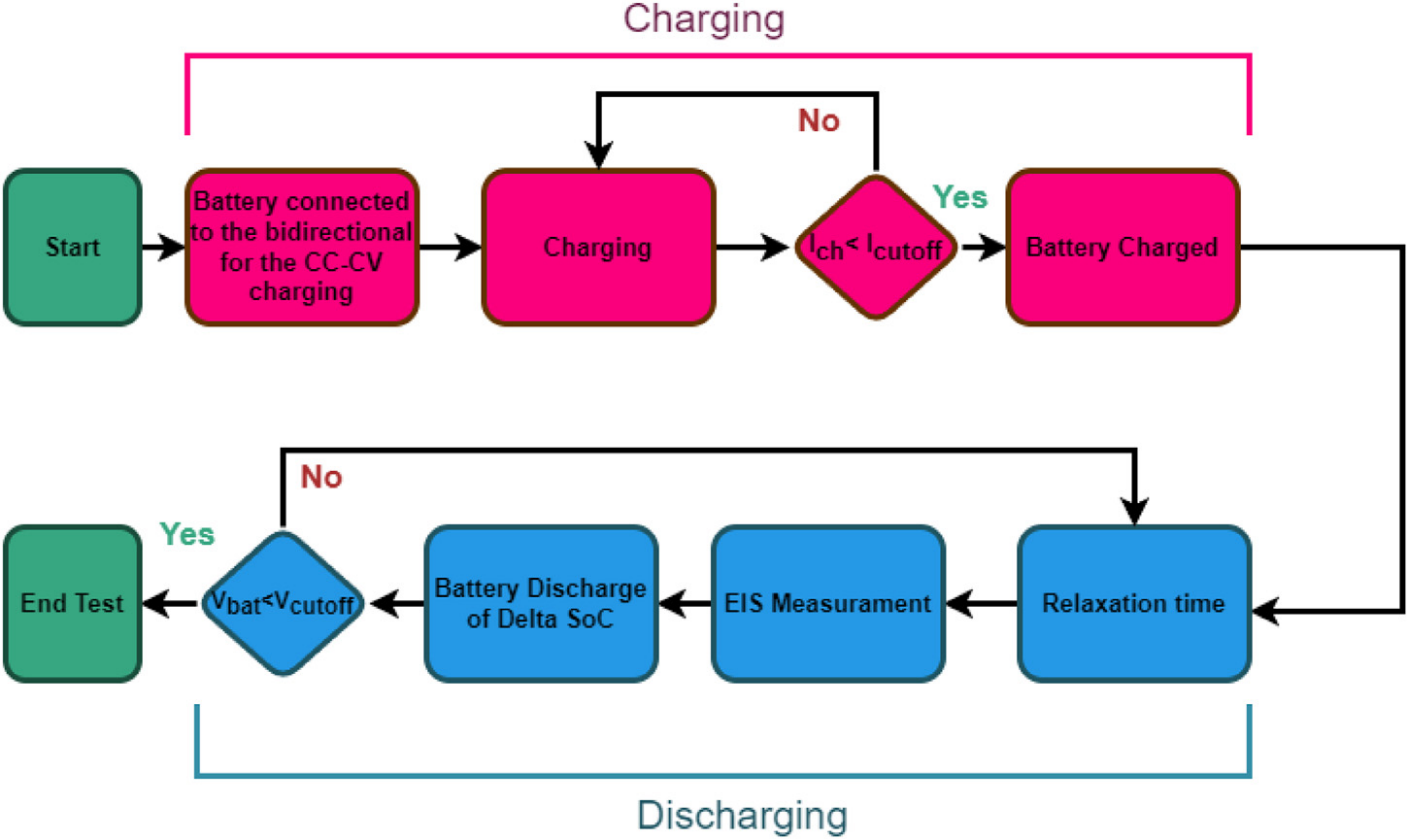
Adopted procedure to obtain the experimental dataset.

EIS measurements were carried out with the Hioki BT4560 instrument, which can force and measure a current flowing into the battery and measure the voltage at the battery terminals. Through the current and voltage measurements, it estimates the impedance of the battery. Specifically, the instrument uses the following root mean square values for the forcing current for different impedance ranges: 150 mA for impedances up to 30 Ω, 50 mA for impedances up to 300 mΩ and 5 mA for impedances up to 3 Ω. This impedance meter allows to measure the battery impedance with stimulus frequencies from 10 mHz to 1 kHz. Since battery impedances are small, the meter uses a four-contact measurement to limit contact and cable resistances. Battery discharging relies on the electronic load Zketech EBD-A20H. This device allows a predefined discharge current to be set up to 20 A. Like the impedance meter, the electronic load has four terminals: two to measure the voltage and two more to impose the discharge current. The developed experimental setup also has a switch system to physically disconnect the electronic load during measurement. This complexity of configuration is necessary for the impedance meter to work properly. A dedicated microcontroller manages the switch system. Each switch has a contact resistance of approximately 100 mΩ. However, this resistance does not affect the discharge current because, as described above, the electronic load has four terminals, allowing the switches' resistance to be compensated. Since battery impedances are small, it is necessary to adopt effective strategies to limit the influence of parasitic phenomena. In detail, the following precautions were used:

• initially, before each test, the geometry of the experimental setup (the position of all the adopted instruments and the connecting cables) was fixed;
• subsequently, whenever the battery under test was changed, the calibration procedure of the impedance meter was carried out. The procedure consists of connecting the meter clips in short circuit and in open circuit to compensate for internal parasitic phenomena within the instrument itself. This operation makes it possible to bring into account, and thus reduce, residual components due to offset and measurement environment;
• finally, as shown in Fig. 3, both the impedance meter and the electronic load are connected to the battery under test via a four-terminal connection. This reduces the influence of contact resistance and connecting cables on the impedance measurement result.

Furthermore, to estimate the measurements quality carried out with the implemented experimental setup, the ISO ENV 13005 “Guide to the Expression of Measurement Uncertainty (GUM)” [21] was followed for the estimation of measurement uncertainties using a Type B evaluation. Specifically, the Hioki BT4560 exhibited a measurement uncertainty lower than 5 mΩ for both the real and imaginary parts of the impedance, while the measurements conducted with the Zketech EBD-A20H had an uncertainty of 0.6 mAh on the capacity measurement.

The whole system is managed by a control software developed in the Python environment. The system requests as input the discharge current, the minimum battery voltage $\left(V_{cut-off}\right)$, the number of impedance measurements, and related stimulus frequencies. The system also outputs the discharge current, voltage data, and impedance measured by the impedance meter. The battery under test is placed in a climatic chamber, where the temperature is kept constant at 20 °C. The chamber uses a Peltier cell, provided by the European Thermodynamic Limited company, to increase or decrease the temperature. According to the manufacturer datasheet, the accuracy of the set temperature is ±1 °C. The chamber is provided with its own temperature sensor placed inside the chamber. Eleven LFP batteries with a nominal capacity of 600 mAh are used as devices under test (DUT); each battery is identified with an identification code. Table 3 shows the main characteristics of the adopted batteries.

**Table 3:** Characteristics of the adopted batteries.

	Battery Characteristics
Manufacturer	O'Cell New Energy Technology CO., LTD
Model	IFR14500EC
Type	Cylindrica
Nominal Capacity	600 mAh
Nominal Voltage	3.2 V
Charge Voltage	3.65 V
Charge Current	300 mA (0.5C)
Cut-off Current	30 mA (0.05C)
Cut-off Voltage	2.0 V

### 4.2 Measurement protocol

Before performing the test, the battery's actual capacity is measured with the Coulomb Counting method. To do it, the battery is placed inside the climatic chamber set to the test temperature. After 10 hours of rest, the battery is deemed to have reached the test temperature. At this stage, the battery is ready for the capacity test, where it is fully charged with the current and manufacture specifications (reported in Table 3) and then discharged with a constant current, which is the same current used for the following test 0.5 C. At the end of this test, the electronic load gives the real capacity of the battery that could be slightly different from the nominal capacity because of inaccuracy in the manufacturing process or materials and calendar aging. The battery's Capacity evaluation is performed once before the discharging-impedance tests because the battery manufacturer predicts a cell cycle life higher than 2000 cycles in test conditions harsher than the one performed here.

Fig. 3 shows the flowchart to obtain a complete test. The protocol is divided into two parts: the battery charging (highlighted in magenta) and the battery discharging (highlighted in blue). The battery charging is performed with the so-called CC-CV methodology. This methodology consists of first a constant charging current (CC) imposed until the battery voltage reaches the maximum voltage ($V_{charge}$), then the voltage is kept constant and equal to the maximum voltage (CV). When the current reaches the threshold value ($I_{cut-off}$), the battery is considered fully charged, and the following discharge test and impedance measurement can be carried out. Batteries, after absorbing or delivering current need a certain time to allow electrochemical recombination processes to occur. This time is referred to as relaxation time. When the battery is charged, the switches are opened, and the electronic load is disconnected. After the relaxation time, equal to one hour, the impedance measurement is performed with all the desired frequencies, and the data are saved to a file on the control PC. After the impedance measurement, the electronic load is reconnected to the battery and discharged of the expected amount of energy (indicated with the ΔSoC parameter) with a C-rate of 0.5 C. This process is iterated for each of the evaluated SoC values, with a 15 minute relaxation time between the discharge and measurement operations. The battery voltage is constantly monitored. If the voltage becomes lower than the minimum battery voltage $\left(V_{cut-off}\right)$, the test stops. The values of $V_{charge}$, $I_{cut-off}$, and $V_{cut-off}$, are provided by the battery manufacturer and reported in Table 3, SoC is defined as the ratio of the amount of energy remaining in the battery to the total energy the battery is capable of delivering at a given SoH. This implies that to know the SoC, the total capacity of the battery should be known. Sometimes, for simplicity, nominal capacity is used, but the energy that the battery is capable of delivering depends on multiple factors such as discharge current, cell temperature, health state, etc. Therefore, within the acquired dataset, there is a discharge test at the test current and temperature without EIS measurements being taken. A Coulomb Counting method, performed directly by the electronic load, gives the actual cell capacity. The ΔSoC parameter is calculated by the battery capacity divided by the total number of SoC levels.

### 4.3 Technical validation

**Fig. 4:**
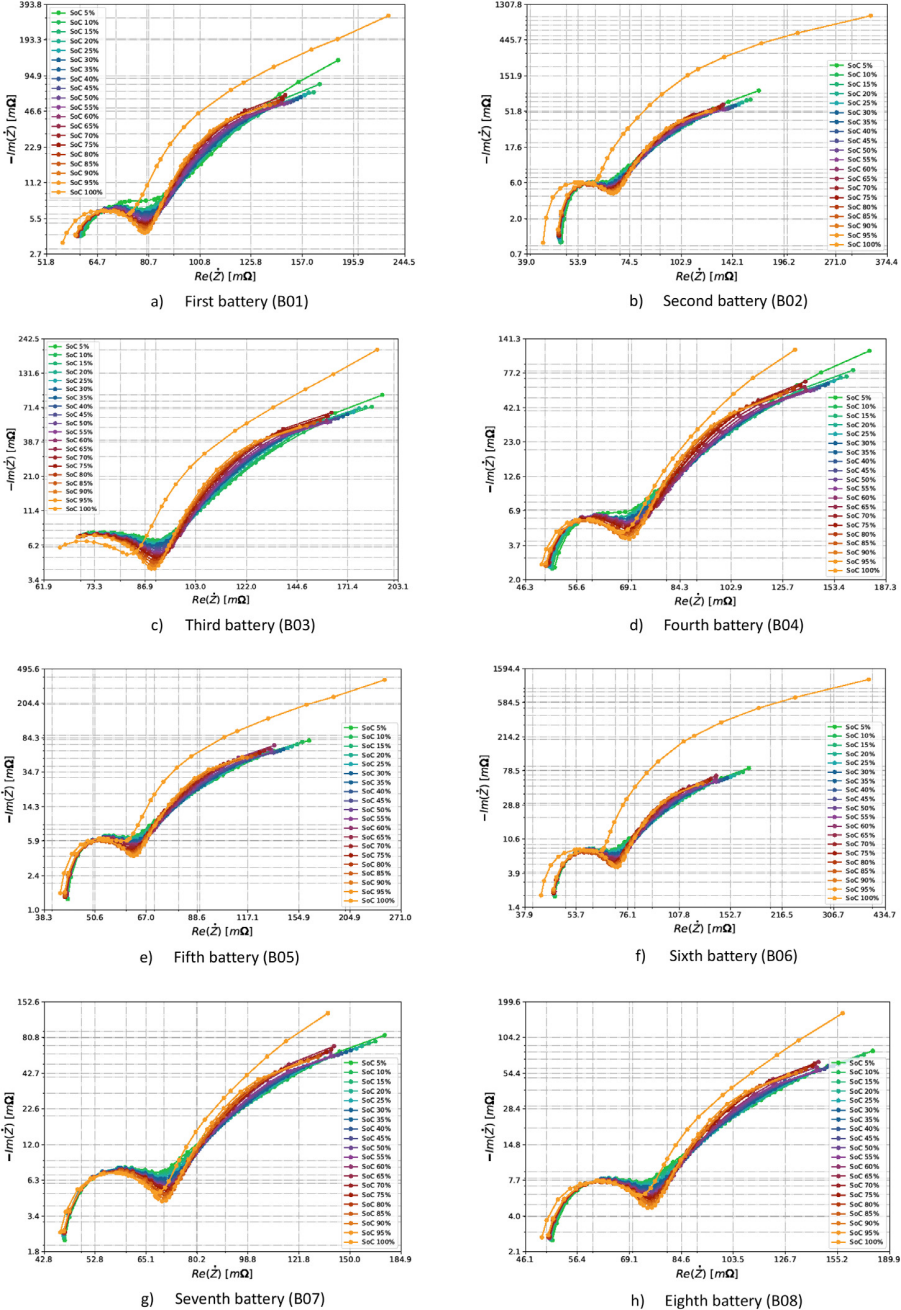

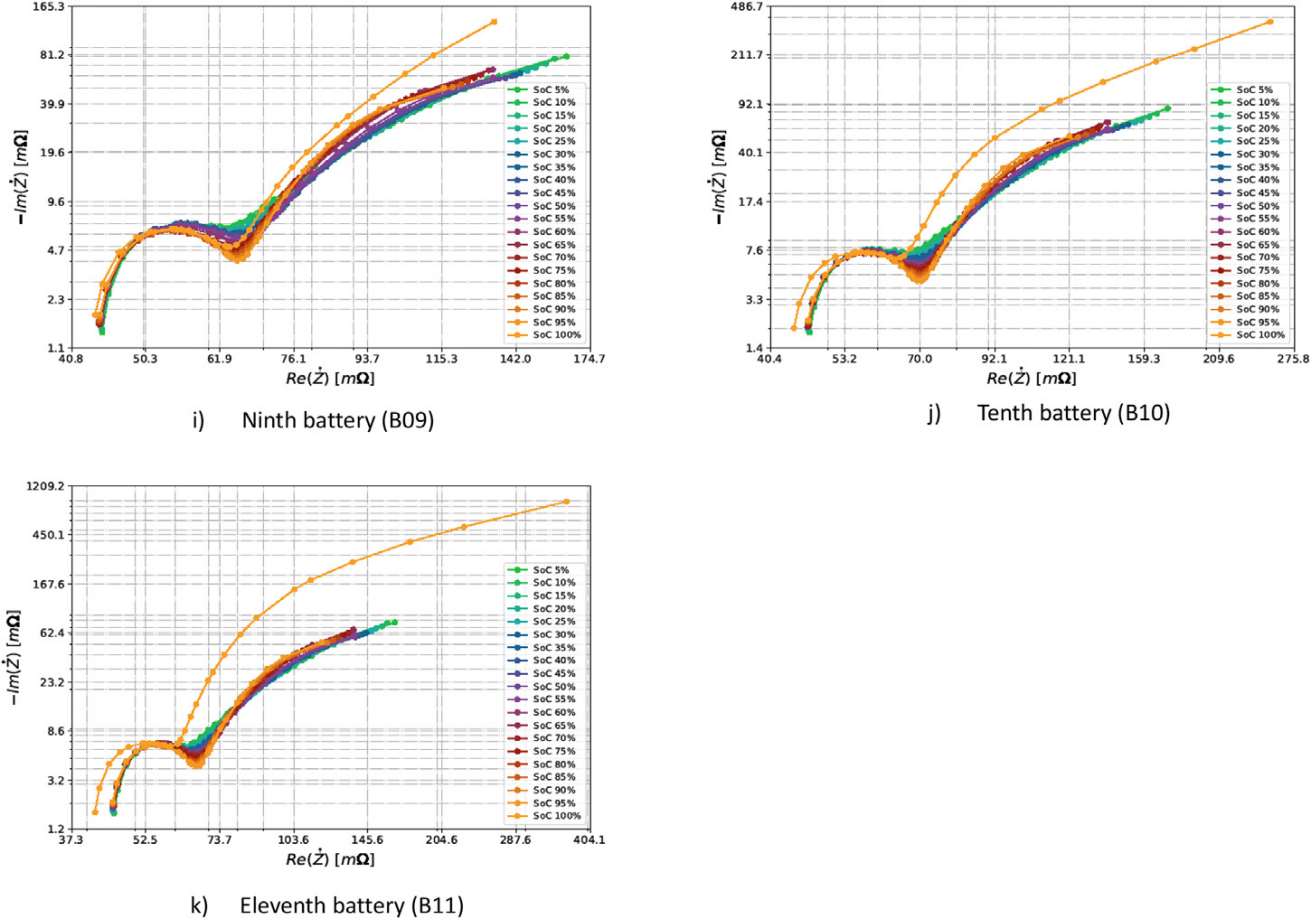
Nyquist plots of the eleven batteries from the first test. The twenty acquired SoC levels are shown for each subfigure.

EIS data are generally represented using Nyquist plots. Analysis using the Nyquist plot allows the identification of outliers in the measurements. The dataset's impedance values were shown through the real component $Re(\dot{Z})$ and the imaginary component $Im(\dot{Z})$. Nyquist plots were created for each SoC level to examine how the LFP batteries' impedance behaved. The measured impedances for the eleven batteries are shown in Fig. 4.

As can be seen in the figures, the measurement with SoC equal to 100% has a very different behavior from the other measurements. This can be explained by analyzing what is happening at the electrochemical level. At high SoC values (close to 100%), the concentration of lithium ions in the positive electrode (cathode) is low [22]. Without lithium ions, the characteristic diffusion times tend to be infinite, and the impedance is higher than other SoCs. Overall, considering the chemistry, type, and capacity of the cells analysed, the results of impedance measurements shown by Nyquist plots are consistent with those described by the scientific community [23]. The impedance range for eleven batteries, as shown in at all SoC levels except SoC 100%, shows similar behavior. However, minor differences are expected due to each battery's unique nature.

### 4.4 Impedance spectra validation

The electrochemical impedance spectroscopy is valid under the small signal hypothesis, or, in other words, when the conditions of linearity, causality, stability, and finiteness are verified. In order to prove these conditions, the Kramer-Kronigs relations and their particular application named “Lin—KK” described in [24,25] are used. In order to apply this method, it is necessary to have a mathematical model of the battery's impedance behavior as the frequency varies. The model estimation is performed by fitting the impedance measurements by varying the frequency with an equivalent circuit model composed of a series of RC elements. An important operation is to determine the number of RC elements necessary to accurately define the impedance behavior. To this end, relative errors committed by the fitting operations on the real part $Re(\dot{Z})$, imaginary part $Im(\dot{Z})$, and complex impedance $\dot{Z}$ are used as a figure of merit to fix the correct number of RC elements. In detail, equations (1)-(3) shows the mathematical definitions of the above relative errors, where N indicates the number of analyzed frequencies, $f_{i}$ is the i-th considered frequency, $Re(\dot{Z}\left(f_{i}\right))$ and $Im(\dot{Z}\left(f_{i}\right))$ represents the measured real and imaginary part for the i-th specific frequency, $Re(\dot{Z}\left(f_{i}\right))$ and $Im(\dot{Z}\left(f_{i}\right))$ represent the estimated real and imaginary part for the i-th specific frequency and $|\dot{Z}|$ is the module of the impedance for the i-th specific frequency.

(1) $$\varepsilon_{Re\left(\dot{Z}\right)}=\sqrt{\sum_{i=1}^{N}{\left(\Delta_{Re}\left(f_{i}\right)\right)}^{2}}=\sqrt{\sum_{i=1}^{N}{\left(\frac{Re\left(\dot{Z}\left(f_{i}\right)\right)-Re\left({\dot{Z}}_{S}\left(f_{i}\right)\right)}{\left|\dot{Z}\left(f_{i}\right)\right|}\right)}^{2}}$$

(2) $$\varepsilon_{Im\left(\dot{Z}\right)}=\sqrt{\sum_{i=1}^{N}{\left(\Delta_{\text{Im}}\left(f_{i}\right)\right)}^{2}}=\sqrt{\sum_{i=1}^{N}{\left(\frac{\text{Im}\left(\dot{Z}\left(f_{i}\right)\right)-\text{Im}\left(\dot{Z}{}_{S}\left(f_{i}\right)\right)}{\left|\dot{Z}\left(f_{i}\right)\right|}\right)}^{2}}$$

(3) $$\begin{array}{ccc} \varepsilon_{\dot{Z}} & = & \sqrt{\sum_{i=1}^{N}{\left(\Delta_{\text{Re}}\left(f_{i}\right)\right)}^{2}+\sum_{i=1}^{N}{\left(\Delta_{\text{Im}}\left(f_{i}\right)\right)}^{2}}\\ & = & \sqrt{\sum_{i=1}^{N}{\left(\frac{\text{Re}\left(\dot{Z}\left(f_{i}\right)\right)-\text{Re}\left({\dot{Z}}_{S}\left(f_{i}\right)\right)}{\left|\dot{Z}\left(f_{i}\right)\right|}\right)}^{2}+\sum_{i=1}^{N}{\left(\frac{\text{Im}\left(\dot{Z}\left(f_{i}\right)\right)-\text{Im}\left({\dot{Z}}_{S}\left(f_{i}\right)\right)}{\left|\dot{Z}\left(f_{i}\right)\right|}\right)}^{2}} \end{array}$$

**Fig. 5:**
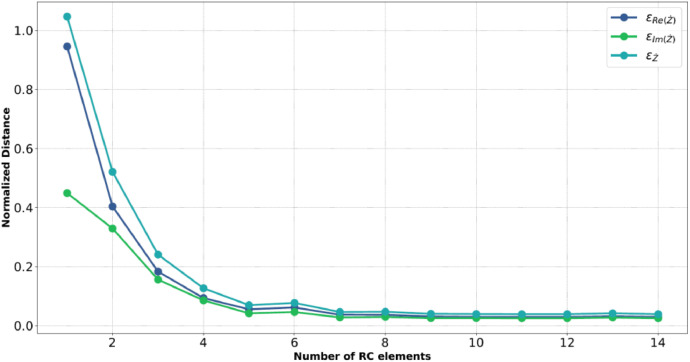
Committed relative errors among the measured and the estimated impedances.

Fig. 5 shows the obtained relative errors by varying the number of considered RC elements for the model estimation operation.

As an example, the Fig. reports the analysis to only one impedance spectra, in particular, the spectra of battery B11 at 20 % of SoC. In particular, we choose to adopt 10 RC elements since from this point on all the relative errors show a very flat behavior with respect to the number of RC elements.

**Fig. 6:**
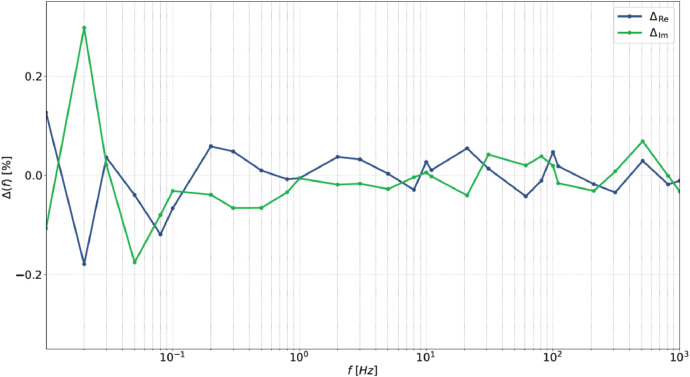
Obtained performance with the Lin-KK method validation.

Once a mathematical model of the battery impedance is created as the frequency changes, it is possible to apply the “Lin—KK” method for the measurement validation. The Lin-KK method is a specific method based on the Kramer Kronig relations and proposed by [25] for the impedance spectra measurement validation. Fig. 6 reports the obtained results for the Lin-KK method. It is possible to note that the estimation performances are always less than 0.3 % for the estimation of both the real and imaginary parts. This confirms the reliability of the conducted analysis.

### 4.5 Machine learning validation

Next, the consistency and reliability of the conducted measurements were verified using a Machine Learning (ML) method, as also shown in [9,26]. The validity test involves the use of the dataset to estimate the SoC. The problem is addressed by a 10-class classification model, where each class represents a 10 % SoC interval. Relevant features, such as real and imaginary component values, were extracted from the dataset, and the data was then split into training and test subsets. The training subset was used to train a ML algorithm based on Support Vector Machines (SVM). The test subset was then used to test the trained model. The ML algorithms' hyperparameters were tuned using the grid search approach. Standard performance measures, such as Accuracy (Acc), Matthews Correlation Coefficient (MCC), Precision (P), Recall (R) and F1 score (F1), were used to evaluate the effectiveness of the ML model. The exact metric definitions are shown in Table 4.

**Table 4:** Definition of metrics for two-class classification problems. They were used in a multiclass problem by averaging the unweighted mean by label.

Acronym	Description	Formula
TP	True Positive is a result where the model correctly predicts the positive class.	/
TN	True Negative is an outcome where the model correctly predicts the negative class.	/
FP	False Positive is an outcome where the model incorrectly predicts the positive class.	/
FN	False Negative is an outcome where the model incorrectly predicts the negative class.	/
Acc	Accuracy is a measure of how well a classification model correctly predicts both the positive and negative classes.	$Acc=\;\frac{TP\;+TN}{TP\;+FP\;+TN\;+FN}$
MCC	The Matthews Correlation Coefficient is a measure that takes into account all confusion matrix values and is particularly useful when dealing with imbalanced datasets.	$MCC=\;\frac{\left(TP\cdot TN)-(FP\cdot FN\right)}{\sqrt{\left(TP+FP)\cdot (TN+FN)\cdot (TN+FP)\cdot (TN+FN\right)}}$
P	Precision is a measure of the model's ability to make accurate positive class predictions.	$Prec=\;\frac{TP\;}{TP\;+FP\;}$
R	Recall measures the model's ability to identify all relevant instances of the positive class.	$Rec=\;\frac{TP\;}{TP\;+FN\;}$
F1	The F1 Score is the harmonic mean of precision and recall. It provides a balance between the two, making it useful when there is a trade-off between these two metrics	$F1=2\cdot \frac{Prec\cdot Rec\;}{Prec+\;Rec\;}$

**Fig. 7:**
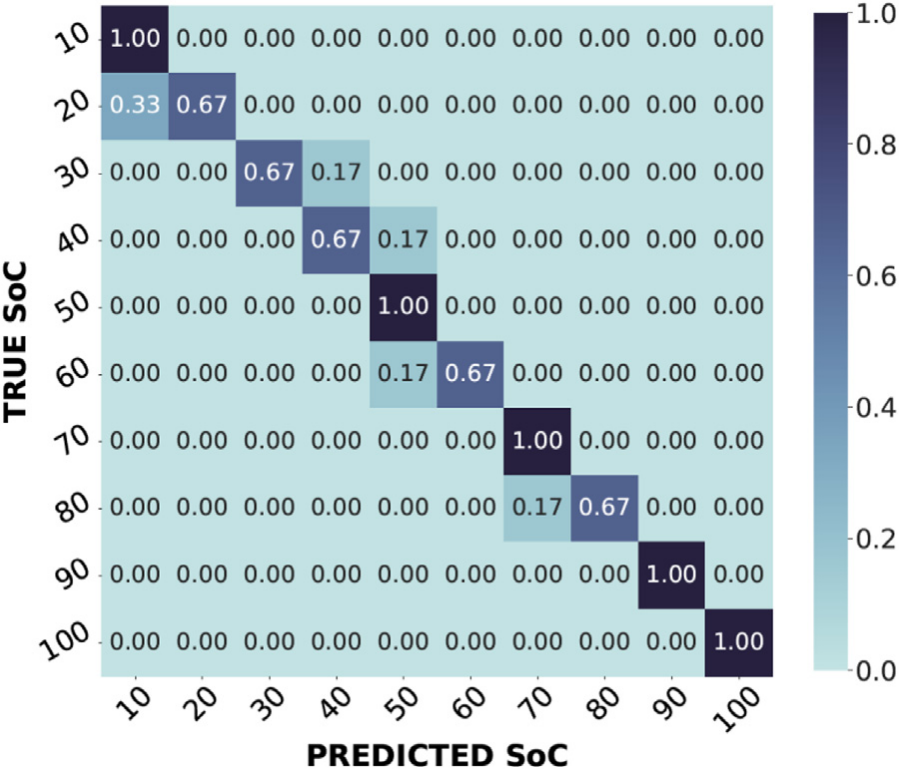
Confusion matrix of the best obtained model.

These metrics are mainly defined for binary classification and have been used in a multiclass problem by averaging the unweighted mean per label. Cross-validation approaches, such as k-fold cross-validation, were used to ensure the robustness and generalisability of the ML model. To reduce the effects of potential data bias or over-fitting, this procedure involves iteratively training and evaluating the models on multiple subsets of the dataset. The predictions from the ML models and the obtained performance further supported the applicability of the dataset for EIS research in the context of SoC estimation. The evaluated hyperparameters and the obtained performances are shown in Table 5, and the confusion matrix in Fig. 7.

**Table 5:** Summary table showing the hyperparameters tested (in bold, those that gave the best performance) and, consequently, the average of the metrics obtained for the best model.

Model	Hyper-parameters	Tested Values	Acc	MCC	P	R	F1
SVM	Regularization parameter (C)	0.1, 1, 10, 100, 1000,10000	0.89	0.88	0.92	0.89	0.89
	Kernel	Linear, polu, rbf, sigmoid					
	Kernel coefficient (gamma)	1,0.1,0.01,0.001,0.0001,scale, auto					
	Decision function shape	one-vs-one, one-vs-rest

### 4.6 Machine learning use case

In this Section, we explore the development of a Machine Learning model to classify different states of charge (SoC) in batteries based on time-series data. The dataset comprises multiple CSV files, each corresponding to a specific SoC level. These files contain critical input features, namely frequency, real component, and imaginary component, which are extracted to form the basis of the classification task. To ensure that each feature set is accurately labelled according to its respective SoC level, we load and process the data in Python. For each CSV file, the input features are systematically extracted, and a unique SoC label is assigned to each set of features based on the source file. These labelled feature sets are then concatenated into a comprehensive dataset, preserving the order and integrity of both the features and their corresponding labels. Once the dataset is consolidated, it is divided into training and testing subsets, adhering to a common practice of allocating approximately 70-80% of the data for training and the remaining 20-30% for testing. This split allows us to build and evaluate a robust classification model. Among the various Machine Learning algorithms, we choose a suitable approach, such as Random Forest, Support Vector Machine (SVM), or a Neural Network-based model, to perform the classification task. The model is trained using the training data, and predictions are made on the test data. The accuracy of these predictions is then compared with the actual SoC labels from the test set, enabling us to assess the model's performance. To evaluate the effectiveness of the model, we calculate key metrics including accuracy, precision, recall, and F1-score. Furthermore, to optimize the model's performance, we employ parameter tuning and optimization techniques. This step is crucial in identifying the most appropriate set of parameters for the chosen Machine Learning algorithm, ultimately leading to more accurate and reliable predictions of the SoC levels. The outcome of this study contributes to advancing the application of machine learning in battery management systems by providing a systematic approach to SoC classification based on time-series data.

### 4.7 Code availability

An example of using the dataset to estimate the SoC is publicly available [27]. In the repository, it is possible to find a Python language project that allows the visualization of the measurements contained in the dataset through Nyquist plots and shows how to train ML algorithms to solve the problem of SoC estimation through classification models.

## Limitations

Not applicable.

## Ethics Statement

The authors have read and follow the ethical requirements for publication in Data in Brief and confirming that the current work does not involve human subjects, animal experiments, or any data collected from social media platforms.

## CRediT authorship contribution statement

**Hamza Mustafa:** Conceptualization, Methodology. **Carmine Bourelly:** Conceptualization, Methodology, Software. **Michele Vitelli:** Conceptualization, Methodology, Software. **Filippo Milano:** Conceptualization, Methodology. **Mario Molinara:** Conceptualization, Methodology. **Luigi Ferrigno:** Conceptualization, Methodology.

## Data Availability

SoC Estimation on Li-ion Batteries: A New EIS-based Dataset for data-driven applications (Original data) (Mendeley Data).
